# Modeling Prognostic Factors in Resectable Pancreatic Adenocarcinomas

**DOI:** 10.4137/cin.s3835

**Published:** 2010-01-20

**Authors:** Taxiarchis Botsis, Valsamo K. Anagnostou, Gunnar Hartvigsen, George Hripcsak, Chunhua Weng

**Affiliations:** 1 Department of Biomedical Informatics, Columbia University, 10032 New York, USA; 2 Department of Computer Science, University of Tromsø, 9037 Tromsø, Norway; 3 Department of Pathology, Yale University School of Medicine, 06511 New Haven, USA.

**Keywords:** pancreatic adenocarcinomas, prognosis, survival, multivariable model

## Abstract

**Background:**

The accurate prognosis for patients with resectable pancreatic adenocarcinomas requires the incorporation of more factors than those included in AJCC TNM system.

**Methods:**

We identified 218 patients diagnosed with stage I and II pancreatic adenocarcinoma at NewYork-Presbyterian Hospital/Columbia University Medical Center (1999 to 2009). Tumor and clinical characteristics were retrieved and associations with survival were assessed by univariate Cox analysis. A multivariable model was constructed and a prognostic score was calculated; the prognostic strength of our model was assessed with the concordance index.

**Results:**

Our cohort had a median age of 67 years and consisted of 49% men; the median follow-up time was 14.3 months and the 5-year survival 3.6%. Age, tumor differentiation and size, alkaline phosphatase, albumin and CA 19-9 were the independent factors of the final multivariable model; patients were thus classified into low (n = 14, median survival = 53.7 months), intermediate (n = 124, median survival = 19.7 months) and high risk groups (n = 80, median survival = 12.3 months). The prognostic classification of our model remained significant after adjusting for adjuvant chemotherapy and the concordance index was 0.73 compared to 0.59 of the TNM system.

**Conclusion:**

Our prognostic model was accurate in stratifying patients by risk and could be incorporated into clinical decisions.

## Introduction

Pancreatic cancer (PC) is estimated to be the fourth leading fatal malignancy for 2009 in US and survival has not improved substantially over the past 30 years.^1^ Pancreatic adenocarcinoma is the most common malignancy of pancreas^2^ and has the worst prognosis among all.^3^ Surgical resection remains the gold standard of care, however only 15%–20% of PC patients are candidates for curative resection.^4^ Prognosis for early stage patients remains poor^3^,^5^ with 5-year survival rates of 25%–30% and 10% for surgically treated lymph node negative and positive patients respectively.^4^

According to the American Joint Committee on Cancer (AJCC) TNM Staging System (6th edition, 2002) a primary pancreatic tumor is considered resectable when limited to the pancreas and/or extending beyond it but without involvement of the celiac axis or the superior mesenteric artery (T_1_, T_2_ and T_3_ pathology staging).^6^ Adjuvant chemotherapy has been shown to improve the outcome of patients with localized pancreatic adenocarcinoma in two multicenter randomized clinical trials;^7^,^8^ however, the most effective chemotherapy regimens and the role of radiation and/or chemo-radiation therapy remain unclear.^9^ The National Cancer Institute (NCI) states that the available data does not resolve the controversy of the optimal adjuvant therapy strategy and suggests that radical pancreatic resection may or may not be combined with postoperative 5-FU chemotherapy and radiation therapy.^10^ Tumour size, lymph node involvement and differentiation have been also reported as the strongest predictors of long-term survival for resected (as this is defined by AJCC) pancreatic adenocarcinoma.^11^–^13^

TNM system does not incorporate prognostic variables other than those defining the TNM stages and factors that could potentially contribute to a more accurate prognostic classification are thus neglected. Here we developed a multivariable prognostic model for resectable pancreatic adenocarcinomas investigating a wide range of clinical and pathological factors.

## Materials and Methods

### Cohort description and data extraction

Cases of pancreatic adenocarcinomas diagnosed between January 1999 and January 2009 were retrospectively retrieved from the NewYork-Presbyterian Hospital/Columbia University Medical Center (New York, NY) clinical data warehouse. A combination of clinical terms (pancreatic tumour and/or carcinoma, adenocarcinoma, Whipple, pancreatectomy, etc) was used for case identification; the extracted records were manually reviewed to confirm their appropriateness for this study. Tumors arising in the duodenum or the peri-ampullary region, patient cases with undefined stage and patients that had received neo-adjuvant therapy before the surgery (stage III at first diagnosis) were excluded; 218 cases of resected pancreatic cancer (stages IA, IB, IIA and IIB) were identified. Patient demographics (age at diagnosis, race, gender), tumor characteristics (localization, size, presence of lymph node metastasis, differentiation), personal medical history (history of other cancer, chronic pancreatitis, cholelithiasis, early onset diabetes mellitus) and history of cancer in first degree relatives, laboratory tests at diagnosis (aspartate aminotransferase-AST, alanine aminotransferase-ALT, alkaline phosphatase-ALP, albumin, total bilirubin) and tumor markers preoperatively (CA 19-9, carcinoembryonic antigen-CEA), surgical resection (totaland distal-pancreatectomy and splenectomy, Whipple resection) are shown in Table 1. Patients were classified according to the AJCC TNM staging system and 8 (4%) stage IA, 16 (7%) stage IB, 36 (17%) stage IIA and 158 (72%) stage IIB tumours were identified (Table 1). The number of metastatic lymph nodes and their percentage over the harvested for the N_1_ patients are also shown in Table 1. This study was approved by the NewYork-Presbyterian Hospital/Columbia University Medical Center Institutional Review Board and was conducted according to the ethical guidelines mandated by the Declaration of Helsinki.

### Statistical analysis

Differences between stages and other factors were investigated using the Kruskal-Wallis statistic for categorical and the t-test statistic for continuous variables. Disease-specific survival was defined as the time from diagnosis (evidenced in the pathology reports) to either death caused by disease or last follow-up. Univariate associations between demographics, clinical characteristics, laboratory values, chemotherapy, surgical treatment and survival were assessed by Cox proportional hazards regression analysis; all variables that were significant at the 0.10 level were further analyzed in a multivariable Cox proportional hazards model. Age at diagnosis and size were analyzed as binary variables split by the median value and the 2 cm cut off point indicated by the TNM staging respectively. Laboratory values were considered either as normal (within reference range) or abnormal (2.5 times the upper limit for AST, ALT, ALP and total bilirubin; below lower limit for albumin; above 200 U/mL for CA 19-9 as suggested by Ferrone et al^14^ and above reference value for CEA). Clinically related variables were examined for interactions prior to further analysis and the data set including the candidate variables identified in univariate analysis was imputed by applying the Multivariate Imputation by Chained Equations (MICE) method assuming that data were missing at random (MAR).^15^ Subsequently, 1000 bootstrap samples were generated based on the imputed set and a backward elimination multivariable Cox proportional hazards model was developed for each bootstrap sample. The Akaike Information Criterion (AIC) was used as the criterion for selection of the best prognostic model for a level of significance of 0.05.^16^ The regression coefficients from the multivariable model were divided with the smallest coefficient in order to calculate a score (equal to the quotient of the division) for each variable, which was then weighted by its coefficient with zero points assigned to the reference category. Subsequently, the scores were summed up into a raw prognostic score and patients were stratified into three risk groups using the tertiles as cut off points for risk classification. Survival curves for the risk groups were constructed using the Kaplan-Meier method and survival differences were analyzed by the log rank test. The prognostic strength of the model was compared to the TNM staging system using the concordance index with 95% confidence intervals.^17^ P values were based on two-sided testing and differences were considered significant at p < 0.05. All statistical analyses were done in R-statistics software (version 2.9.1); the Kaplan-Meier curves were constructed in SPSS (version 15.0 for Windows, Chicago, IL).

## Results

### Patient characteristics

Two-hundred and eighteen patients diagnosed with early stage pancreatic ductal adenocarcinoma who underwent surgical resection were identified; our cohort consisted of 107 men (49%) and 111 women (51%) with a median age of 64.0 (range: 42–86, mean ± SE: 64.0 ± 1.0) and 70.0 (36–88, mean ± SE: 67.9 ± 1.0) respectively. One-hundred and sixty-seven (76.6%) patients were white, 14 (6.4%) black and 17 (7.8%) and 18 (8.3%) were of Hispanic and Asian origin respectively; race for two patients had not been recorded. Thirteen patients (6%) reported family history of breast and/or ovarian cancer, and 11 (5%) of pancreatic cancer in first degree relatives; of the latter four patients reported a pair of affected first degree relatives. Thirty-three (15%) patients had personal history of cancer. The surgical treatment for the treatment of the primary tumor involved total pancreatectomy and splenectomy for 5 (3%), distal pancreatectomy and splenectomy for 24 (11%), and Whipple resection for 189 (86%) patients. One hundred and nine patients (50%) received adjuvant chemotherapy. The median length of follow up for all patients was 14.3 months (range: 0.7–118.7, mean ± SE: 18.5 ± 1.1) with a 5-year, 3-year and 1-year disease specific survival of 3.6%, 12.7% and 66.7% respectively.

Presence of nodal metastases was identified in 157 (72.0%). The majority of adenocarcinomas arose in the head/neck (n = 184, 84.4%) and less in the body/tail (n = 34, 15.6%). Nineteen (8.7%) of the tumors were poorly, 81 (37.2%) moderately and 111 (50.9%) well differentiated; differentiation was missing for 7 (3.2%) cases. There was no difference between stage I and stage II patients with respect to age (median age at diagnosis 69.5 years, range: 54–83, mean ± SE: 68.3 ± 1.8 and 67.0 years, range: 36–88, mean ± SE: 66.1 ± 0.8 respectively, p = 0.34), gender (p = 0.1), tumour differentiation (p = 0.67) or personal history of other cancer (p = 0.36). All clinical and pathological characteristics are summarized in Table 1.

### Identification of predictors of survival

Age at diagnosis over 67 years (HR: 1.83, 95% CI: 1.33–2.50, p < 0.001), presence of lymph node metastasis (HR: 1.50, 95% CI: 1.05–2.16, p = 0.027), tumor size over 2 cm (HR: 1.89, 95% CI: 1.24–2.91, p = 0.003) and personal history of other cancer (HR: 1.61, 95% CI: 1.04–2.51, p = 0.033; Table 2) were associated with worse prognosis; well and moderately differentiated tumors had significantly better outcome compared to poorly differentiated tumors (HR = 0.38, 95% CI: 0.20–0.72, p = 0.003 and HR = 0.63, 95% CI: 0.45–0.89, p = 0.008 respectively; Table 2). High levels of ALP (HR: 1.68, 95% CI: 1.09–2.59, p = 0.019), low levels of albumin (HR: 2.17, 95% CI: 1.27–3.69, p = 0.004) and CA 19-9 ≥ 200 U/mL (HR: 1.53, 95% CI: 1.04–2.28, p = 0.033) were also poor prognostic factors. Additionally, black race had worse prognosis compared to the white race (HR: 1.94, 95% CI: 1.07–3.53, p = 0.029).

Univariate analysis showed that adjuvant chemotherapy added significant benefit to the disease outcome (HR: 0.45, 95% CI: 0.32–0.62, p < 0.001; Table 2); there was no difference between stage I and stage II (n = 12 and n = 97, respectively; p = 0.99) or stage IA, IB, IIA and IIB (n = 3, n = 9, n = 17 and n = 80, respectively; p = 0.83) patients who received adjuvant chemotherapy. The number of metastatic lymph nodes binarized by the median value (equals 3) and their percentage over the total number of harvested nodes (split at 25%) was further analyzed and patients with more than 3 infiltrated lymph nodes had worse prognosis (HR = 1.51, 95% CI: 1.04–2.19; Table 2). Whipple procedure was not associated with better outcome compared to pancreatectomy (total or distal) and splenectomy (HR: 1.09, 95% CI: 0.66–1.82, p = 0.72; Table 2). TNM stages were associated with survival such that only patients with stage IIB had an increased risk compared to stage IA patients (HR = 3.39, 95% CI: 1.24–9.24, p = 0.017; Table 2). Survival rates were similar among patients with IA, IB and IIA disease (log rank p_(IA vs. IB)_ = 0.125, log rank p_(IA vs. IIA)_ = 0.056 and log rank p_(IB vs. IIA)_ = 0.718); also, differences were not observed between stage IIB and stage IIA (log rank p = 0.311) and IB (log rank p = 0.315) respectively. However, the broader grouping of patients into stages I (n = 24) and II (n = 194) showed more distinct outcome with stage II patients having worse survival (HR = 1.82, 95% CI: 1.08–3.07, p = 0.025).

### Development of a multivariable prognostic model

Variables significantly correlated with survival in univariate analysis at a level of significance 0.10 were further incorporated in a multivariate Cox proportional hazards regression analysis using a stepwise selection/backward elimination process on each of the 1000 bootstrap samples. No interactions between the clinically related variables were observed. Age at diagnosis (HR = 1.40, 95% CI: 1.00–1.95, p = 0.045), tumor differentiation (HR = 0.65, 95% CI: 0.50–0.83, p < 0.001), size (HR = 1.72, 95% CI: 1.11–2.67, p = 0.016), alkaline phosphatase (HR = 1.59, 95% CI: 1.05–2.43, p = 0.029), albumin (HR = 2.35, 95% CI: 1.38–4.03, p = 0.002), and CA 19-9 (HR = 1.49, 95% CI: 1.05–2.11, p = 0.027) were the independent prognostic factors that were included in the final model (Table 3).

Based on the factor coefficients we developed a prognostic scoring system assigning 1 point to age over 67 years, 1.5 points to tumor size over 2 cm, 1.5 points to high ALP, 1 point to CA 19-9 over 200 U/mL, 2.5 points to low albumin, and −1.5 points and −3 points to moderately and well differentiated tumors respectively. All scores were summed up into a raw prognostic score and tertiles were used as cut off points to classify patients into three groups. Scores ranged from (−3)–0, 0.5–4 and 4.5–7.5 for the low (n = 14), intermediate (n = 124) and high (n = 80) risk groups respectively.

**Figure 1.**
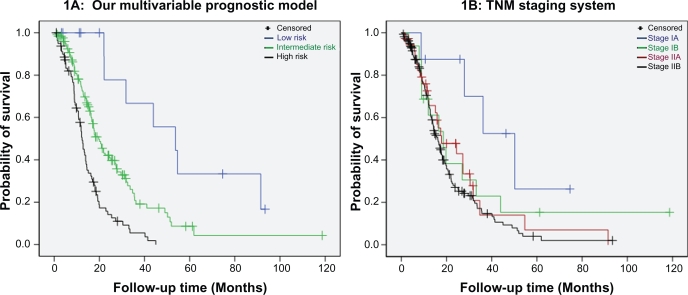
Disease outcome by **A**) our multivariable model with low risk patients showing an exceptional benefit towards survival compared to intermediate (log rank p = 0.005) and high risk patients (log rank p < 0.001); intermediate vs. high risk patients also have better outcome (log rank p < 0.001) and **B**) the AJCC TNM staging system (log rank p_(IA vs. IB)_ = 0.125, p_(IA vs. IIA)_ = 0.056, p_(IA vs. IIB)_= 0.010, p_(IB vs. IIA)_ = 0.718, p_(IB vs. IIB)_ = 0.315 and p_(IIA vs. IIB)_ = 0.311).

Patients in the low risk group had a better prognosis compared to patients in the intermediate (median survival 53.7 vs. 19.7 months, p = 0.005; Fig. 1A) and high risk group (median survival 53.7 vs. 12.3 months, p < 0.001; Fig. 1A). Patients classified in the intermediate risk group showed a distinct benefit towards overall survival compared to patients of the high risk group (median survival 19.7 vs. 12.3 months, p < 0.001; Fig. 1A). Interestingly 4 and 7 patients with IIA and IIB TNM disease respectively were classified in the low risk group whereas 7 stage IB patients were classified in the high risk group, underlying the weakness of the AJCC TNM system to accurately stratify patients by risk based on the tumor characteristics only (survival curves for AJCC TNM stages are shown in Fig. 1B). The concordance index calculated to assess the accuracy of our model was equal to 0.73 (95% CI: 0.58–0.84). The concordance index of the TNM staging system was equal to 0.59 (95% CI: 0.42–0.74) when patients were grouped into stages IA, IB, IIA and IIB and 0.62 (95% CI: 0.38–0.81) when they were classified into stages I and II.

Subsequently, we adjusted our final model for adjuvant chemotherapy by performing a second multivariable Cox proportional hazard analysis. The prognostic stratification of our model (HR = 3.91, 95% CI: 1.72–8.85, p = 0.001 and HR = 9.37, 95% CI: 4.02–21.83, p < 0.001 for the intermediate and high risk groups respectively) and adjuvant chemotherapy (HR = 0.38, 95% CI: 0.27–0.53, p < 0.001) were both significantly correlated with survival indicating that our prognostic classification is an independent predictor of outcome for resectable pancreatic ductal adenocarcinoma. The benefit of adjuvant chemotherapy did not differ between low, intermediate and high risk patients who received chemotherapy treatment (n = 7, n = 66 and n = 36, respectively; p = 0.52).

## Discussion

The TNM staging system classifies patients with exocrine pancreatic cancer according to their pathologic characteristics and distinguishes between localized resectable (Stages I and II), locally advanced (Stage III) and metastatic disease (stage IV).^6^ The outcome for patients undergoing surgical resection cannot be accurately predicted based on the TNM classification alone highlighting the need for incorporation of other parameters in such systems. We developed a robust multivariable model for stratifying resectable pancreatic ductal adenocarcinomas by risk and compared its prognostic accuracy with the TNM staging system. Our model classified patients with higher accuracy compared to the TNM system; concordance indexes were equal to 0.73 and 0.59 respectively.

Patients classified as stage II by the TNM system had a shorter survival compared to stage I patients (HR = 1.82, 95% CI: 1.08–3.07, p = 0.025), however the only significant pairwise comparison among TNM subgroups was between Stages IA and IIB (p = 0.017; Fig. 1B). Survival rates among patients in any other subgroup did not differ significantly and this highlights the inherent weaknesses of the current staging system. Our prognostic score stratified patients in three groups with clear outcome and significant survival differences were found for all pairwise comparisons.

We found that ALP is an independent prognostic factor for resectable pancreatic ductal adenocarcinomas; this has not been shown before to the best of our knowledge. ALP has been shown to be a significant independent factor for advanced pancreatic cancer^18^ and that a 1.8-fold increase of ALP was associated with poor prognosis in a veterans’ cohort, independently of tumor stage.^19^ Serum albumin was strongly correlated with survival in our multivariable model; this is consistent with the report of Schnelldorfer et al who found that preoperative low albumin was a negative predictor of survival in patients with pancreatic adenocarcinoma undergoing a Whipple procedure; however they failed to prove its independent prognostic value.^3^

We demonstrated that pre-operative CA 19-9 level above 200 U/mL was an important independent prognostic factor. The selection of the cut off point was based on the study of Ferrone et al who examined CA 19-9 levels as a prognostic factor for patients with resectable pancreatic adenocarcinoma and suggested 200 U/mL as the appropriate cut-off point.^14^ Additionally, recent studies showed that CA 19-9 elevation over the laboratory reporting limit (>37 U/mL) may occur in patients without malignant disease.^20^,^21^ In light of these findings and of our results we suggest the shift of the critical CA 19-9 values to a higher limit; 200 U/mL could be a reliable choice.

Tumor differentiation and size have been shown to be independent prognostic factors for patients with resectable pancreatic adenocarcinoma in various studies.^2^,^13^,^22^–^25^ Nevertheless, only Winter et al followed the same AJCC criteria with us to define a pancreatic tumor as resectable.^13^ Also, the split for tumor size varied among these studies with the criterion of 2 cm being selected in two cases only.^23^,^25^ Age at diagnosis was included in our multivariable model and this is an important finding given the controversy around the prognostic potential of age for resectable ductal adenocarcinomas (summarized by Garcea et al).^26^

Patients receiving adjuvant chemotherapy had a clear survival benefit in both univariate and multivariate analyses in our population which is consistent with the findings of Neoptolemos et al^7^ and Oettle et al.^8^ The benefit was similar for all patients receiving adjuvant chemotherapy independently of their grouping into the TNM stages or our own groups. Most importantly our prognostic classification was independent of adjuvant chemotherapy and this is the first report of such a model to the best of our knowledge.

Potential limitations of the current study are the retrospective nature of data collection and the imputation of variables with missing values. Nevertheless, it should be mentioned that data imputation has been proven to be superior to both complete case analysis and missing-indicator method in multivariable diagnostic research^27^ and has been suggested as the ideal approach to address missingness in retrospective analyses.^28^–^30^ Also, MICE method is indicated for the imputation of categorical variables with specific variables acting as predictors in this process.^15^

It could be also argued that the lack of validation with an external independent data set reduces the impact of our work. However, the selected bootstrap resampling approach supports the construction of validated predictive models, especially when it is combined with automated variable selection methods.^16^,^31^ Doubtless, the validation of our model in a prospectively collected cohort could potentially establish it in clinical practice.

In conclusion, we developed an accurate multivariable prognostic model for resectable pancreatic ductal adenocarcinomas, incorporating age, tumor differentiation and size, preoperative CA 19-9, serum albumin and alkaline phosphatase. The AJCC TNM staging system that classifies patients based on tumor characteristics only was found to be inferior compared to our model. Our results indicate that the addition of prognostic factors other than the traditional tumorrelated ones could lead to a more accurate prognostic stratification of patients with resectable pancreatic ductal adenocarcinoma. Such an approach could dramatically improve clinical decision-making.

## Figures and Tables

**Table 1.** Cohort characteristics.

**Variable**	**No. of patients**
**Age at diagnosis**	
≤67 years	112
>67 years	106
**Race**	
White	167
Black	14
Hispanic	17
Asian	18
Not recorded	2
**Gender**	
Female	111
Male	107
**Stage**	
Stage IA	8
Stage IB	16
Stage IIA	36
Stage IIB	158
**Pathology T**	
T_1_	13
T_2_	42
T_3_	163
**Pathology N**	
N_0_	61
N_1_	157
Metastatic lymph nodes*	
≤3	80
>3	75
Missing	2
Percentage of metastatic lymph nodes*	
≤25%	78
>25%	77
Missing	2
**Differentiation**	
Poor	111
Moderate	81
Well	19
Missing	7
**Localization**	
Proximal pancreas (head/neck)	184
Distal pancreas (body/tail)	34
**Tumour size**	
≤2 cm	41
>2 cm	173
Missing	4
**Smoking**	
Never	62
Former/Current	75
Missing	81
**Alcohol**	
Never	106
Former/Current	22
Missing	90
**Chronic Pancreatitis**	
No	74
Yes	144
**History of other cancer**	
No	149
Yes	33
Missing	36
**Cholelithiasis**	
No	190
Yes	28
**Diabetes mellitus**	
No	124
Yes	60
Missing	34
**AST**	
Normal	136
Abnormal	63
Missing	19
**ALT**	
Normal	135
High	69
Missing	14
**Alkaline phosphatase**	
Normal	174
Abnormal	31
Not recorded	13
**Albumin**	
Normal	28
Abnormal	177
Missing	13
**Total bilirubin**	
Normal	169
Abnormal	36
Missing	13
**CA 19-9**	
<200 U/mL	112
≥200 U/mL	53
Missing	53
CEA	
Normal	89
High	77
Missing	52
**Chemotherapy**	
No adjuvant chemotherapy	109
Adjuvant chemotherapy	109
**Surgery**	
Pancreatectomy (distal or total) and splenectomy	29
Whipple procedure	189
Family history**	
Breast, ovarian cancer	13 (1)
Pancreatic cancer	11 (4)
Gastrointestinal (Other)	8 (0)
Other types	17 (3)
Not contributory	52
Missing	122

* Split at the median.

** At least 1st degree relative (pairs of 1st degree relatives in parenthesis).

**Table 2.** Results of the Univariate Cox proportional hazards regression analysis.

**Variable**	**HR (95% CI)**	**p-value**
**Age at diagnosis**		<0.001
≤67 years	1	
>67 years	1.83 (1.33–2.50)	
**Race**		0.129
White	1	
Black	1.94 (1.07–3.53)	0.029
Hispanic	1.08 (0.58–2.01)	0.808
Asian	1.39 (0.80–2.42)	0.245
**Gender**		0.304
Female	1	
Male	0.85 (0.62–1.16)	
**Stage**		0.069
Stage IA	1	
Stage IB	2.41 (0.78–7.50)	0.128
Stage IIA	2.72 (0.94–7.89)	0.065
Stage IIB	3.39 (1.24–9.24)	0.017
**Pathology T**		0.114
T_1_	1	
T_2_	2.32 (1.03–5.21)	0.042
T_3_	2.20 (1.02–4.73)	0.044
**Pathology N**		0.027
N_0_	1	
N_1_	1.50 (1.05–2.16)	
Metastatic lymph nodes*		0.029
≤3	1	
>3	1.51 (1.04–2.19)	
Percentage of metastatic lymph nodes*		0.151
≤25%	1	
>25%	1.31 (0.91–1.88)	
**Differentiation**		0.001
Poor	1	
Moderate	0.63 (0.45–0.89)	0.008
Well	0.38 (0.20–0.72)	0.003
**Localization**		0.768
Proximal pancreas (head/neck)	1	
Distal pancreas (body/tail)	0.93 (0.59–1.47)	
**Tumour Size**		0.003
≤2 cm	1	
>2 cm	1.89 (1.24–2.91)	
**Smoking**		0.253
Never	1	
Former/Current	1.28 (0.84–1.94)	
**Alcohol**		0.366
Never	1	
Former/Current	1.31 (0.73–2.35)	
**Chronic pancreatitis**		0.132
No	1	
Yes	1.29 (0.93–1.80)	
**History of other cancer**		0.033
No	1	
Yes	1.61 (1.04–2.51)	
**Cholelithiasis**		0.296
No	1	
Yes	0.77 (0.47–1.26)	
**Diabetes mellitus**		0.509
No	1	
Yes	1.13 (0.79–1.62)	
**AST**		0.282
Normal	1	
Abnormal	1.21 (0.85–1.72)	
**ALT**		0.726
Abnormal	1	
High	0.94 (0.67–1.33)	
**Alkaline phosphatase**		0.019
Normal	1	
Abnormal	1.68 (1.09–2.59)	
**Albumin**		0.004
Normal	1	
Abnormal	2.17 (1.27–3.69)	
**Total bilirubin**		0.943
Normal	1	
Abnormal	1.02 (0.67–1.55)	
**CA 19-9**		0.033
<200 U/mL	1	
≥200 U/mL	1.53 (1.04–2.28)	
**CEA**		0.358
Normal	1	
High	1.19 (0.82–1.71)	
**Chemotherapy**		<0.001
No adjuvant chemotherapy	1	
Adjuvant chemotherapy	0.45 (0.32–0.62)	
**Surgery**		0.723
Pancreatectomy (distal or total) and Splenectomy	1	
Whipple procedure	1.09 (0.66–1.82)	

* For patients with pathology N_1_ status.

**Table 3.** Multivariable analysis results of Cox proportional hazards regression model.

	**Variable**	**Coefficient**	**HR (95% CI)**	**p-value**
**Prognostic model** (AIC = 1373.749)	Age at Diagnosis	0.338	1.40 (1.00–1.95)	0.045
	Differentiation	−0.435	0.65 (0.50–0.83)	< 0.001
	Tumour size	0.543	1.72 (1.11–2.67)	0.016
	Alkaline Phosphatase	0.467	1.59 (1.05–2.43)	0.029
	Albumin	0.856	2.35 (1.38–4.03)	0.002
	CA 19-9	0.397	1.49 (1.05–2.11)	0.027

## References

[1] Jemal A Siegel R Ward E Hao Y Xu J Thun MJ Cancer statistics CA Cancer J Clin 2009 59 225 49 19474385 10.3322/caac.20006

[2] Sohn TA Yeo CJ Cameron JL Resected adenocarcinoma of the pancreas-616 patients: results, outcomes, and prognostic indicators J Gastrointest Surg 2000 4 567 79 11307091 10.1016/s1091-255x(00)80105-5

[3] Schnelldorfer T Ware AL Sarr MG Long-term survival after pancreatoduodenectomy for pancreatic adenocarcinoma: is cure possible? Ann Surg 2008 247 456 62 18376190 10.1097/SLA.0b013e3181613142

[4] Zuckerman DS Ryan DP Adjuvant therapy for pancreatic cancer: a review Cancer 2008 112 243 9 18050292 10.1002/cncr.23174

[5] Labianca R Berardi E Malugani F Challenges in the treatment of gastrointestinal tumours Ann Oncol 2006 17 Suppl 5 v137 41 16807443 10.1093/annonc/mdj969

[6] Greene FL American Joint Committee on Cancer American Cancer Society. AJCC cancer staging handbook from the AJCC cancer staging manual New York Springer 2002

[7] Neoptolemos JP Stocken DD Friess H A randomized trial of chemoradiotherapy and chemotherapy after resection of pancreatic cancer N Engl J Med 2004 350 1200 10 15028824 10.1056/NEJMoa032295

[8] Oettle H Post S Neuhaus P Adjuvant chemotherapy with gemcitabine vs. observation in patients undergoing curative-intent resection of pancreatic cancer: a randomized controlled trial JAMA 2007 297 267 77 17227978 10.1001/jama.297.3.267

[9] Saif MW Adjuvant treatment of pancreatic cancer in 2009: where are we? Highlights from the 45th ASCO annual meeting. Orlando, FL, USA. May 29–June 2 JOP 2009 10 373 7 19581737

[10] NCI. Stage I and II Pancreatic Cancer. 2009

[11] Goh BK Tan YM Cheow PC Outcome of distal pancreatectomy for pancreatic adenocarcinoma Dig Surg 2008 25 32 8 18292659 10.1159/000117821

[12] Howard TJ Krug JE Yu J A margin-negative R0 resection accomplished with minimal postoperative complications is the surgeon’s contribution to long-term survival in pancreatic cancer J Gastrointest Surg 2006 10 1338 45; discussion 1345 6 17175452 10.1016/j.gassur.2006.09.008

[13] Winter JM Cameron JL Campbell KA 1423 pancreaticoduodenectomies for pancreatic cancer: A single-institution experience J Gastrointest Surg 2006 10 1199 210; discussion 1210 1 17114007 10.1016/j.gassur.2006.08.018

[14] Ferrone CR Finkelstein DM Thayer SP Muzikansky A FernandezdelCastillo C Warshaw AL Perioperative CA 19-9 levels can predict stage and survival in patients with resectable pancreatic adenocarcinoma J Clin Oncol 2006 24 2897 902 16782929 10.1200/JCO.2005.05.3934 PMC3817569

[15] van Buuren S Boshuizen HC Knook DL Multiple imputation of missing blood pressure covariates in survival analysis Stat Med 1999 18 681 94 10204197 10.1002/(sici)1097-0258(19990330)18:6<681::aid-sim71>3.0.co;2-r

[16] Austin PC Tu JV Bootstrap methods fof developing predictive models The American Statistician 2004 52 131 7

[17] Harrell FE Jr Califf RM Pryor DB Lee KL Rosati RA Evaluating the yield of medical tests JAMA 1982 247 2543 6 7069920

[18] Stocken DD Hassan AB Altman DG Modelling prognostic factors in advanced pancreatic cancer Br J Cancer 2008 99 883 93 19238630 10.1038/sj.bjc.6604568 PMC2538756

[19] Siddiqui A Heinzerling J Livingston EH Huerta S Predictors of early mortality in veteran patients with pancreatic cancer Am J Surg 2007 194 362 6 17693283 10.1016/j.amjsurg.2007.02.007

[20] Bedi MM Gandhi MD Jacob G Lekha V Venugopal A Ramesh H CA 19-9 to differentiate benign and malignant masses in chronic pancreatitis: is there any benefit? Indian J Gastroenterol 2009 28 24 7 19529898 10.1007/s12664-009-0005-4

[21] Kim HR Lee CH Kim YW Han SK Shim YS Yim JJ Increased CA 19-9 level in patients without malignant disease Clin Chem Lab Med 2009 47 750 4 19402792 10.1515/CCLM.2009.152

[22] Benassai G Mastrorilli M Quarto G Factors influencing survival after resection for ductal adenocarcinoma of the head of the pancreas J Surg Oncol 2000 73 212 8 10797334 10.1002/(sici)1096-9098(200004)73:4<212::aid-jso5>3.0.co;2-d

[23] Meyer W Jurowich C Reichel M Steinhauser B Wunsch PH Gebhardt C Pathomorphological and histological prognostic factors in curatively resected ductal adenocarcinoma of the pancreas Surg Today 2000 30 582 7 10930222 10.1007/s005950070096

[24] Ueda M Endo I Nakashima M Prognostic factors after resection of pancreatic cancer World J Surg 2009 33 104 10 19011933 10.1007/s00268-008-9807-2

[25] Yeo CJ Cameron JL Lillemoe KD Pancreaticoduodenectomy for cancer of the head of the pancreas. 201 patients Ann Surg 1995 221 721 31; discussion 731 3 7794076 10.1097/00000658-199506000-00011 PMC1234702

[26] Garcea G Dennison AR Pattenden CJ Neal CP Sutton CD Berry DP Survival following curative resection for pancreatic ductal adenocarcinoma. A systematic review of the literature JOP 2008 9 99 132 18326920

[27] van der Heijden GJ Donders AR Stijnen T Moons KG Imputation of missing values is superior to complete case analysis and the missing-indicator method in multivariable diagnostic research: a clinical example J Clin Epidemiol 2006 59 1102 9 16980151 10.1016/j.jclinepi.2006.01.015

[28] Arnold AM Kronmal RA Multiple imputation of baseline data in the cardiovascular health study Am J Epidemiol 2003 157 74 84 12505893 10.1093/aje/kwf156

[29] Clark TG Altman DG Developing a prognostic model in the presence of missing data: an ovarian cancer case study J Clin Epidemiol 2003 56 28 37 12589867 10.1016/s0895-4356(02)00539-5

[30] Clark TG Stewart ME Altman DG Gabra H Smyth JF A prognostic model for ovarian cancer Br J Cancer 2001 85 944 52 11592763 10.1054/bjoc.2001.2030 PMC2375096

[31] Hastie T Tibshirani R Friedman JH The elements of statistical learning: data mining, inference, and prediction2nd edn New York Springer 2009

